# Mechanical Ventilation Enhances HMGB1 Expression in an LPS-Induced Lung Injury Model

**DOI:** 10.1371/journal.pone.0074633

**Published:** 2013-09-10

**Authors:** Ning Ding, Fang Wang, Hui Xiao, Lixin Xu, Shouzhang She

**Affiliations:** 1 Department of Anesthesiology, Guangzhou First People’s Hospital, Guangzhou Medical University, Guangzhou, China; 2 Department of Medicine, Shandong Binzhou Vocational College, Binzhou, China; 3 Department of Out-patient, Guangzhou First People’s Hospital, Guangzhou Medical University, Guangzhou, China; University of Giessen Lung Center, Germany

## Abstract

**Background:**

Mechanical ventilation (MV) can augment inflammatory response in lipopolysaccharide (LPS) challenged lungs. High mobility group box 1 protein (HMGB1) is a pro-inflammatory mediator in ventilator-induced lung injury, but its mechanisms are not well defined. This study investigated the role of HMGB1 in lung inflammation in response to the combination of MV and LPS treatment.

**Methods:**

Forty-eight male Sprague-Dawley rats were randomized to one of four groups: sham control; LPS treatment; mechanical ventilation; mechanical ventilation with LPS treatment. Mechanically ventilated animals received 10 ml/kg tidal volumes at a rate of 40 breaths/min for 4 h. In the HMGB1-blockade study, sixteen rats were randomly assigned to HMGB1 antibody group or control antibody group and animals were subjected to MV+LPS as described above. A549 cells were pre-incubated with different signal inhibitors before subjected to 4 h of cyclic stretch. Lung wet/dry weight (W/D) ratio, total protein and IgG concentration, number of neutrophils in bronchoalveolar lavage fluid (BALF), and lung histological changes were examined. The levels of interleukin-1β (IL-1β), IL-6, tumor necrosis factor-α (TNF-α), macrophage inflammatory protein-2 (MIP-2) and HMGB1 in BALF were measured using ELISA. Real-time quantitative PCR and Western blot were used to analyze mRNA and protein expression of HMGB1. Western blot were employed to analyze the activation of IκB-α, NF-κB, JNK, ERK, and p38.

**Results:**

MV significantly augmented LPS-induced lung injury and HMGB1 expression, which was correlated with the increase in IL-1β, IL-6 and MIP-2 levels in BALF. *In vivo,* intratracheally administration of HMGB1 antibody significantly attenuated pulmonary inflammatory injury. *In vitro* experiments showed cyclic stretch induced HMGB1 expression through signaling pathways including p38 and NF-κB.

**Conclusions:**

The findings indicated that moderate tidal volume MV augmented LPS induced lung injury by up-regulating HMGB1. The mechanism of HMGB1-mediated lung injury is likely to be signaling through p38 and NF-κB pathways.

## Introduction

Despite its life-saving potential, mechanical ventilation (MV) may initiate or augment acute lung injury (ALI), which is recognized as ventilator-induced lung injury (VILI) [1]–[5]. Although moderate tidal volume (VT) alone does not seem sufficient for lung injury, many studies have shown that it may augment pre-existing lung injury [6]–[9]. It is thought that the additional insult, or second “hit” induced by MV, synergizes with the underlying inflammatory process, resulting in detrimental effects on the lung [10]–[12].

One of the underlying mechanisms of VILI is the release of pro-inflammatory cytokines, such as tumor necrosis factor (TNF)-α, interleukin (IL)-1β and macrophage inflammatory protein (MIP)-2, in response to MV associated mechanical stretch [13], [14]. In clinical practice, treatment aimed to limit the initial inflammatory state has not proven successful [15]. However, limiting the second “hit” caused by MV may represent a viable therapy. High mobility group box 1 protein (HMGB1) has recently been proposed as a potent inflammatory mediator in ALI [16]. The biological activities of HMGB1 include activation of macrophages/monocytes, upregulation of endothelial adhesion molecules, stimulation of epithelial cell barrier failure, and mediation of fever and anorexia [16]. Intratracheal administration of HMGB1 has been found to induce acute lung injury [17]. Furthermore, an increase in HMGB1 level in response to MV has been observed recently in both animal experiment and clinical trial [18], [19]. Importantly, blocking HMGB1 led to a significant reduction in lung inflammatory reaction [19]. Our recent study demonstrated that cyclic stretch significantly increased HMGB1 expression in pulmonary alveolar epithelial cells, which was correlated with the elevated levels of TNF-α, IL-1β and IL-6 [20].

A number of studies conducted recently demonstrated that purified HMGB1 had no proinflammatory activity and only acted as a chemoattractant and a mitogen. Instead, it bounds pathogen-associated molecules, such as LPS and IL-1β, enhanced the cytokine effects of these molecules [21]–[25]. Thus, HMGB1 has dual activities, solo or in company, which may serve our body’s necessity to sacrifice or reconstruct tissues as required by the presence or absence of pathogens. In the current study, we utilized *in vitro* and *in vivo* models of VILI to test the hypothesis that HMGB1 induced by mechanical ventilation dose not produce pro-inflammatory activity, but may interact with LPS or cytokines and potentiate their pro-inflammatory effects. The findings indicated that moderate tidal volume MV may increase the severity of lung injury by up-regulating HMGB1 at a phase where LPS challenging is present.

## Materials and Methods

### Animals

A total of sixty-four male Sprague-Dawley rats (weighing 250–300 g) were included in the experiment. Forty-eight animals were prospectively randomized to one of four groups (n = 12 per group): spontaneous breath (sham); spontaneous breath with LPS treatment (LPS); mechanical ventilation (MV); mechanical ventilation with LPS treatment (MV+LPS). In the HMGB1-blockade study, sixteen rats were randomly and evenly assigned to HMGB1 antibody group or control antibody group and animals were then subjected to MV+LPS. The study was approved by the Animal Care and Use Committee of Guangzhou Medical University. Animals were handled in accordance with the national guide for care and use of laboratory animals. All surgery was performed under anesthesia, and all efforts were made to minimize suffering.

### Experimental Protocol

Animals were anesthetized with intraperitoneal injection of pentobarbital sodium (60 mg/kg) and ketamine (80 mg/kg), anesthesia was maintained by infusion of pentobarbital at 15 mg/kg every 30 minutes via the tail vein, and muscle relaxation was maintained with pancuronium (2 mg/kg/h). The trachea was exposed and a 16-gauge catheter was inserted. A right carotid artery catheter (PE-50 tubing, BD biosciences, Franklin Lakes, NJ, USA) was cannulated for continuous monitoring mean arterial pressure (Digi-Med BPA-400, Micro-Med, Louisville, KY, USA) and gas analysis (i-STAT 300, Abbott, Princeton, NJ, USA). Animals received an intravenous injection of LPS (Escherichia coli O111: B4; Sigma, St. Louis, MO, USA) at a dose of 5 mg/kg or saline solution. Rats were then randomized to MV (10 ml/kg tidal volume, no PEEP, 40 breaths/min), with room air (FiO_2_ = 21%) for 4 h with a small animal respirator (Model Inspira, Harvard Apparatus, South Natick, MA, USA) or spontaneous breath. Peak inspiratory pressure (PIP) was recorded at the beginning and at the end of the MV period. At the end of the experiment, animals were sacrificed and half of the right lung was immediately snap-frozen and stored at −80°C until further analysis, the rest right lung was intratracheally instilled with 10% formalin and excised for histological evaluation. The left lung was taken for measurement of lung wet/dry weight ratios.

### HMGB1 Antibody Treatment

In the HMGB1-blockade study, sixteen rats were randomly and evenly assigned to HMGB1 antibody group or control antibody group, and received intratracheally 2 mg of anti-HMGB1 neutralizing antibody (Shino-test, Sagamihara, Kanagawa, Japan) or nonspecific isotype-specific antibody IgY (Shino-test, Sagamihara, Kanagawa, Japan), respectively. Animals were subjected to MV+LPS as described above 1 h later. The dose of anti-HMGB1 antibody that inhibits HMGB1 release *in vivo* was determined in a pilot experiment.

### Cell Mechanical Stretch

Human lung epithelial cells (A549) were cultured and subjected to cyclic stretch (CS) using a FX-4000T system (Flexercell, McKeesport, PA, USA) as previously described [26]. A CS of 20% strain at 30 cycles/min was applied for 4 hours. When necessary, cells were pre-incubated with SB203580 (p38 MAPK inhibitor), PD98059 [extracellular signal-regulated kinase (ERK) inhibitor], SP600125 [c-Jun N-terminal kinase (JNK) inhibitor] (Cell Signaling, Danvers, MA, USA), SN-50 (NF-κB inhibitor, Calbiochem, San Diego, CA, USA) or vehicle dimethyl sulfoxide (DMSO).

### Lung Wet/Dry Weight Ratio

Lung wet weight was determined immediately after removal and the lungs were then placed in an oven at 75°C for 48 hours and reweighed. The wet/dry weight ratio was calculated as the ratio of the wet weight to the dry weight.

### Histological Examination

The right lung was serially sectioned, embedded in paraffin, and stained with hematoxylin and eosin (H&E). A modified lung injury histological scoring system was applied, based on the following pathological features: alveolar congestion; hemorrhage; neutrophil margination and tissue infiltration; and thickness of the alveolar wall. A score of 0 represented normal lung, and scores of 1, 2, 3, and 4 represented mild (<25%), moderate (25–50%), severe (50–75%), and very severe (>75%) lung involvement, respectively [27].

### Bronchoalveolar Lavage

At the conclusion of the experiment, the left lungs were lavaged three times with 2 mL of cold saline, and the bronchoalveolar lavage fluid (BALF) was collected. Differential cell counts were done using Wright-Giemsa staining. Total protein concentration was measured with bicinchoninic acid protein assay (Pierce, Rockford, IL, USA) and IgM concentration was measured with enzyme-linked immunosorbent assay (ELISA, Bethyl Laboratory, Montgomery, TX, USA).

### Lung Myeloperoxidase Activity

Lung myeloperoxidase (MPO) activity was determined by ELISA according to the manufacturer’s instructions (Cell Sciences, Canton, MA, USA).

### Measurement of Cytokines

Quantikine ELISA kits specific for rats IL-1β, IL-6, TNF-α, MIP-2 (R&D Systems, Minneapolis, MN, USA) and HMGB1 (IBL, Hamburg, Germany) were used to determine the cytokines concentrations in BALF according to the manufacturer’s instructions.

### Western Blot Analysis

Proteins were extracted from lung tissues or cells and the concentrations were determined. Equivalent amounts of protein per well were separated on sodium dodecyl sulfate polyacrylamide gel and then electrotransferred to polyvinylidine fluoride membrane under semi-dry conditions. After blocking with 5% skimmed milk, membranes were incubated with primary antibody against HMGB1 (Abcam, Cambridge, MA, USA), IκB-α, p-IκB-α, p65 NF-κB, JNK, p-JNK, ERK, p-ERK, p38 and p-p38 (Cell Signaling, Danvers, MA, USA) and subsequently with a secondary horseradish peroxidase conjugated antibody. The bands were detected using West Pico enhanced chemiluminescence kit (Thermo Fisher Scientific, Rockford, IL, USA). Membranes were stripped and reprobed for β-actin (Abcam, Cambridge, MA, USA), which served as a loading control.

### Reverse Transcription and Real-Time Polymerase Chain Reaction

Total RNA was extracted from lung tissues using RNeasy Mini Kit (Qiagen, Valencia, CA, USA). iScript™ reverse transcription supermix for RT-qPCR kit (Bio-Rad, Hercules, CA, USA) was used for reverse transcription. PCR amplification mixtures were prepared using iTaq™ Fast SYBR Green Supermix with ROX (Bio-Rad, Hercules, CA, USA) and Real-time PCR was performed with MX3000p (Stratagene, La Jolla, CA, USA). Quantification of gene expression was calculated relative to β-actin.

### Statistical Analysis

Data are expressed as means±standard deviation (SD). Statistical differences were assessed using Student’s *t*-test, Mann-Whitney rank sum test, or one-way analysis of variance (ANOVA), followed by the Fisher LSD post hoc test or by the Holm-Sidak method, where appropriate. The strength of correlations was calculated by Pearson’s analysis. Significance was established at p<0.05.

## Results

### Induction of Lung Injury

All animals survived the experiment. There were no significant differences in PIP, PaO_2_/FiO_2_ ratio and arterial pH before the induction of MV among groups. In the MV+LPS group, PIP significantly increased and PaO_2_/FiO_2_ ratio decreased after 4 h of MV (p<0.05). PH analysis revealed a significantly decreased pH in MV+LPS group at the end of MV (p<0.05) (Table 1).

**Table 1:** Comparison of PIP and arterial blood gases between two groups.

	Group	MV	
		0 h	4 h
PIP	MV	12.5±1.6	15.1±2.0
	MV+LPS	13.2±1.9	29.6±3.1*
PaO_2_/FiO_2_	MV	447.5±32.3	402.7±29.1
	MV+LPS	438.6±35.2	243.3±27.2*
pH	MV	7.43±0.06	7.41±0.07
	MV+LPS	7.40±0.08	7.26±0.15*

* p<0.05 vs 0 h.

**Figure 1:**
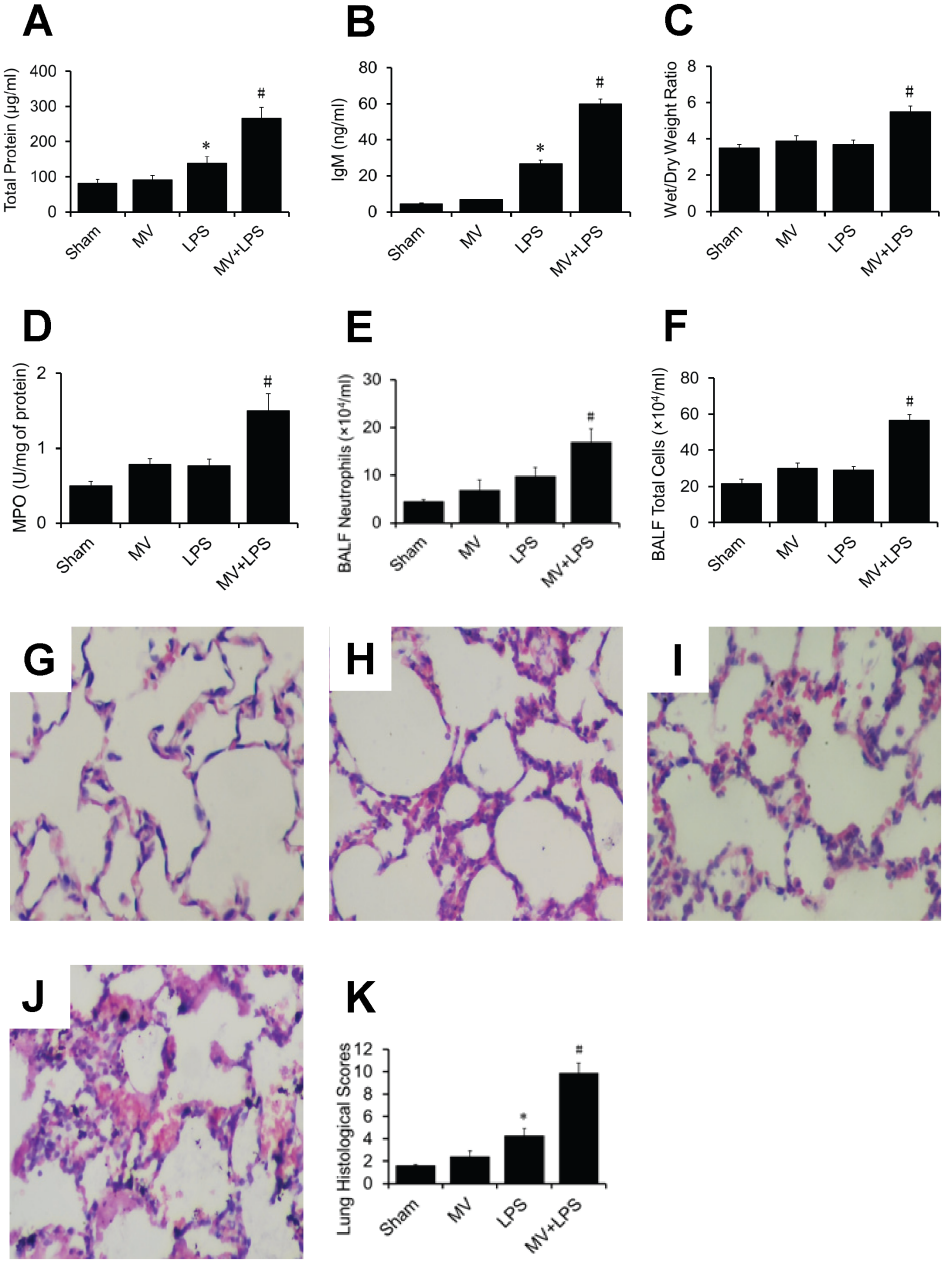
Effect of MV and LPS on lung inflammatory injury. Concentrations of total protein (A) and IgM (B) in BALF, wet/dry lung weight ratio (C), lung MPO activity (D), neutrophils (E) and total cell numbers (F) were measured from the four groups of rats exposed to sham maneuver, MV, LPS and MV+LPS. Sections from the left lung lobe were stained with hematoxylin and eosin. Representative photos are shown for each experimental group (original magnification × 400). Note that lung sections from sham group (G) showed a normal alveolar structure, MV group (H) and LPS group (I) showed mild inflammatory changes (I), MV+LPS group (J) showed pronounced leukocyte infiltration and increased septal thickening. Lung injury histological scores (K). Data shown are means±SD from 6 rats per group. *, p<0.05 vs sham group; #, p<0.05 vs LPS group.

Total protein (Fig. 1A), IgM (Fig. 1B), wet/dry lung weight ratio (Fig. 1C), MPO activity (Fig. 1D), neutrophils (Fig. 1E) and total cells (Fig. 1F) in MV+LPS group increased significantly compared with sham control (p<0.05). H&E staining revealed normal lung histology in sham rats (Fig. 1G). Mild inflammatory changes were observed in lung tissues from MV group and LPS group (Fig. 1H, 1I). In MV+LPS group, interstitial edema and inflammatory cell infiltration were markedly increased (Fig. 1J). Quantitative analysis showed a significant increase of lung injury score in MV+LPS group (Fig. 1K).

### HMGB1 Expression and Cytokines Production

**Figure 2:**
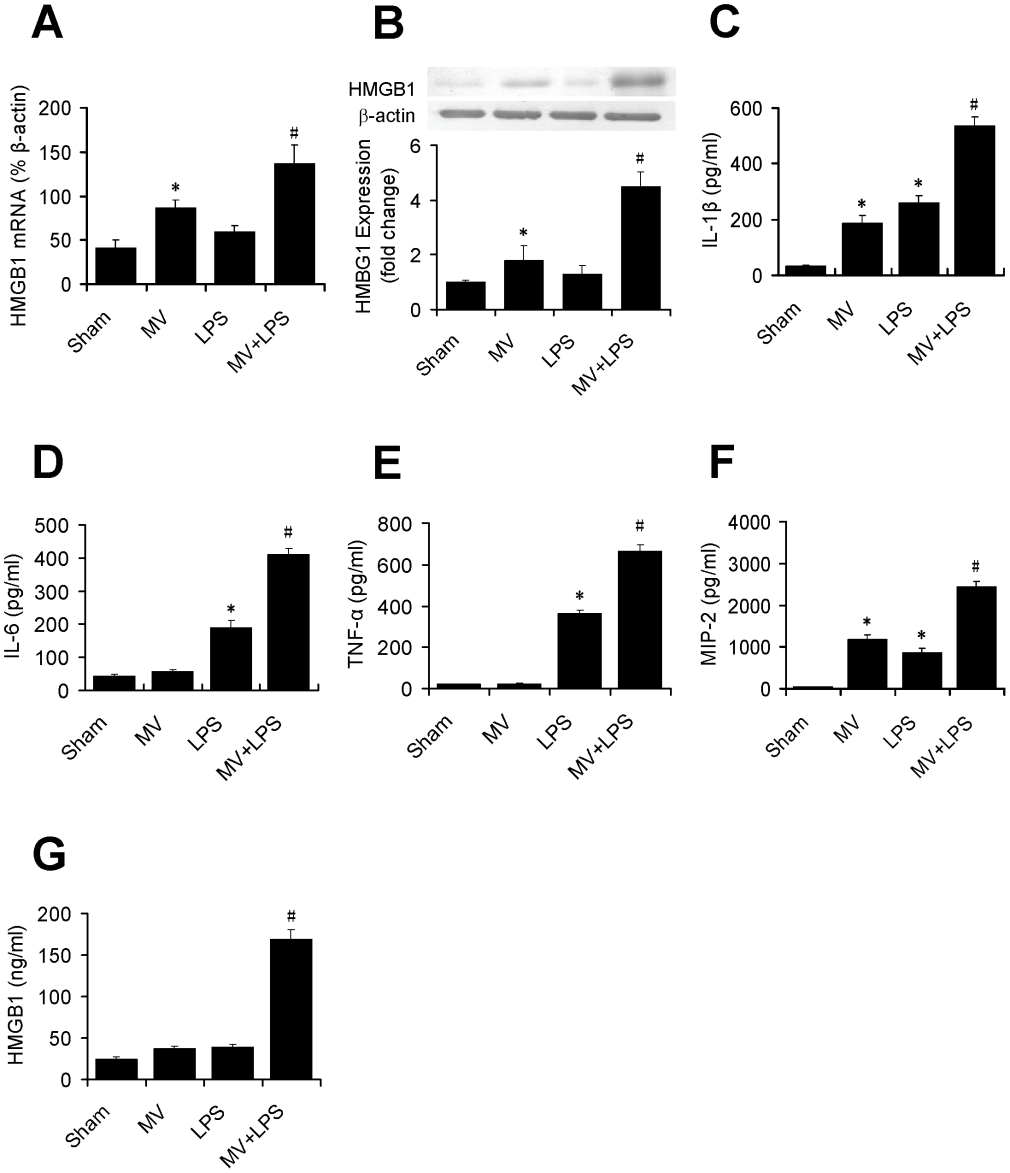
Effect of MV and LPS on HMGB1 expression and cytokines production. Quantitative RT-PCR was performed with total RNA from each animal, and HMGB1 mRNA expression in the lung was shown as percentage over β-actin gene in the same sample (A). HMGB1 protein expression was determined by Western blotting in the lung and the intensity ratio of HMGB1 to β-actin for each band is shown (B). ELISA was performed for protein levels of IL-1β (C), IL-6 (D), TNF-α (E), MIP-2 (F) and HMGB1 (G) in BALF from each animal. Data shown are means±SD from 6 rats per group. *, p<0.05 vs sham group; #, p<0.05 vs LPS group.

Compared with sham group, HMGB1 mRNA and protein levels in MV group significantly increased (2.08 fold and 1.79 fold, respective). Interestingly, LPS alone enhanced HMGB1 expression but did not reach statistical significance. However, when combined with MV, LPS markedly induced HMGB1expression (Fig. 2A, 2B).

IL-1β, IL-6, TNF-α and MIP-2 in BALF significantly increased in LPS group, and IL-1β and MIP-2 increased in MV group compared with sham group. Interestingly, there was a synergistic augmentation of all four cytokines when treated with both mechanical ventilation and LPS (Fig. 2C–2F). There was no significant effect of MV or LPS alone on HMGB1 level in BALF, however, MV and LPS synergistically increased HMGB1 concentration in BALF (Fig. 2G). Correlation analysis revealed that in MV+LPS group, the upregulation of IL-1β, IL-6 and MIP-2 were in positive correlation with HMGB1 (p = 0.047, r = 0.54; p = 0.022, r = 0.65 and p = 0.021, r = 0.89, respectively). The correlation between TNF-α and HMGB1 did not reach significance (p = 0.23, r = 0.33). No such correlations were found in the other groups.

### Activation of p38 and NF-κB

**Figure 3:**
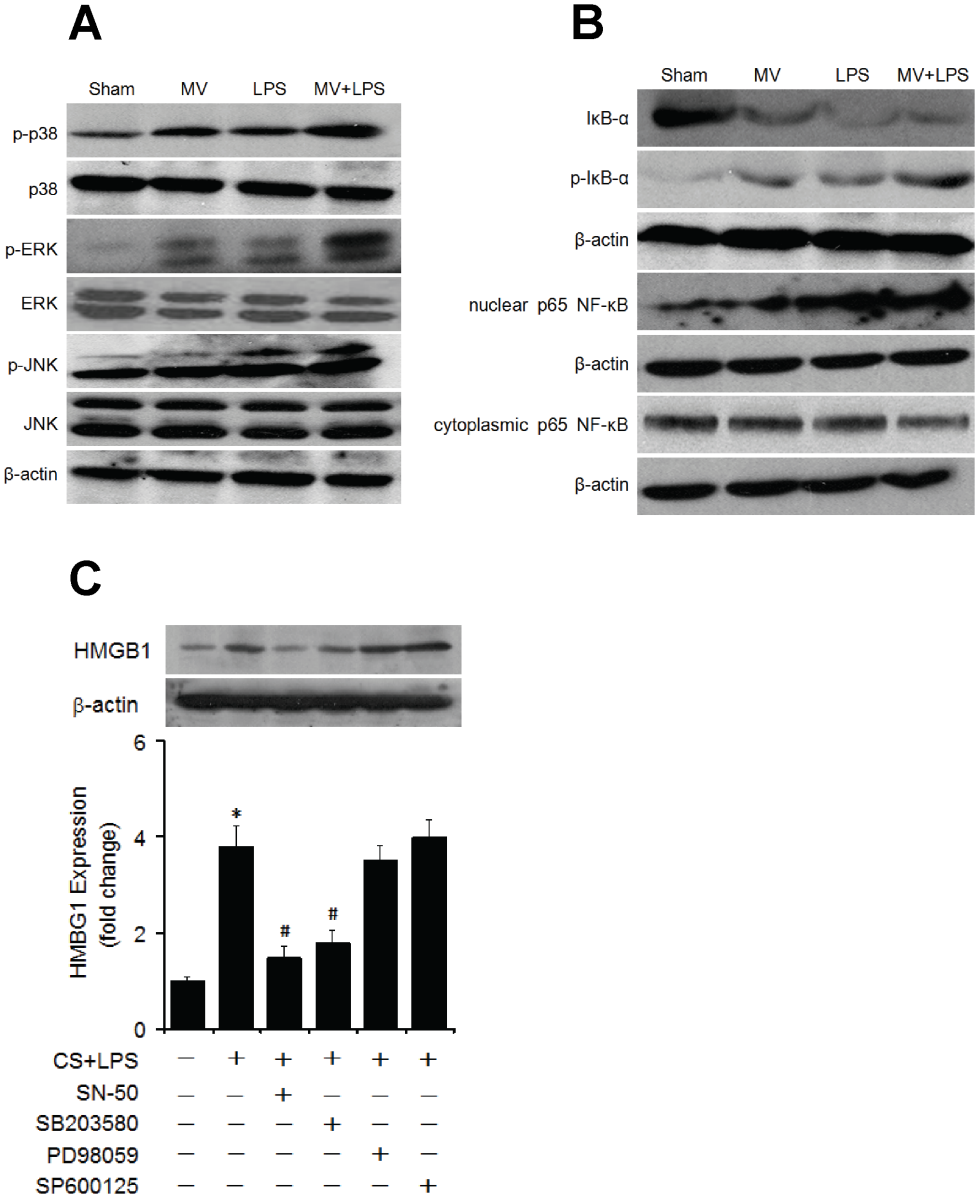
Involvement of p38 MAPK and NF-κB signaling in MV and LPS induced lung injury. Sham and LPS-treated rats randomized to either spontaneous respiration or moderate tidal volume mechanical ventilation (10 ml/kg) for 4 h. The proteins from lung tissues were used for the detection of p38, ERK, JNK (A) or IκB-α and p65 subunit of NF-κB in the nucleus and cytoplasm (B). Human lung epithelial cells (A549) were pre-incubated with NF-κB inhibitor SN-50, p38 inhibitor SB203580, ERK1/2 inhibitor PD98059 or JNK inhibitor SP 600125 followed by cyclic stretch (CS) at 20% strain, 30 cycle/min for 4 hrs. Cells were then lysed and protein was extracted for Western blot analysis (C). Note remarkable inhibition of HMGB1 expression in animals with SN-50 or SB203580 treatment, but not PD98059 or SP600125. β-actin was used as the loading control. Representative blots of three experiments are shown. All data are expressed as mean±SD. *, p<0.05 vs control group; #, p<0.05 vs CS+LPS group.

Western blot analysis indicated that both MAPKs and NF-κB pathways were significantly activated after LPS or MV treatment. Additionally, MV significantly increased the LPS-dependent activation of these pathways (Fig. 3A, 3B). SN-50 and SB203580 significantly inhibited HMGB1 expression, however, PD98059 and SP600125 did not show the inhibiting effect (Fig. 3C).

### Inhibition of HMGB1 Prevents VILI

**Figure 4:**
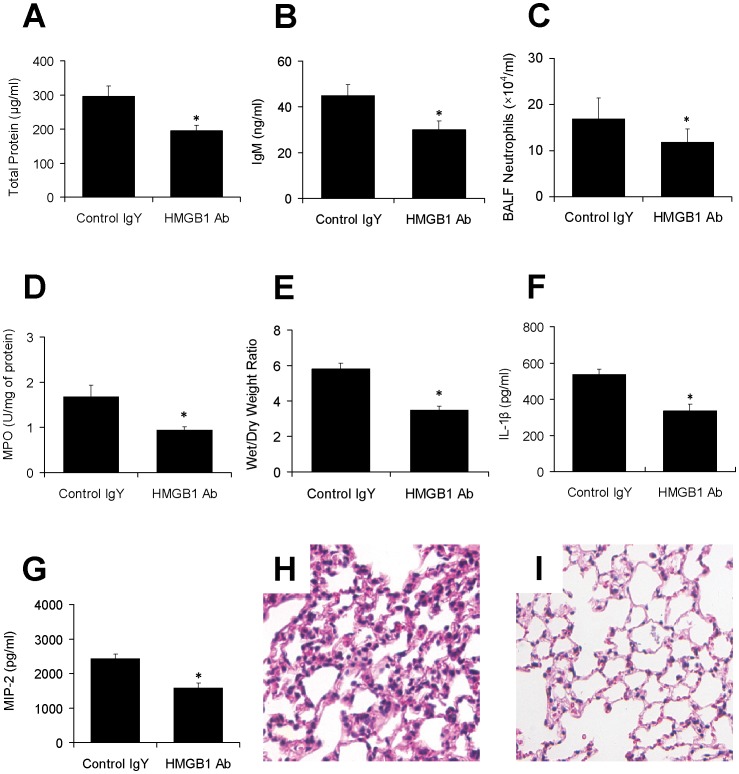
Effect of HMGB1 blocking antibody on ventilator-induced lung injury in rats. Animals received anti-HMGB1 blocking antibody intratracheally before challenged by LPS and MV. BALF concentrations of total protein (A) and IgM (B), neutrophils (C), lung MPO activity (D), lung wet/dry weight ratio (E), IL-1β (F) and MIP-2 (G) concentrations, and histopathology (H, I) were measured. H: MV+LPS; I: MV+LPS+ HMGB1 antibody. Data are expressed as mean±SD. *, p<0.05 vs IgY group.

Compared with control IgY, HMGB1 antibody significantly attenuated the increase of total protein (Fig. 4A) and IgM (Fig. 4B) concentrations, neutrophils (Fig. 4C), MPO activity (Fig. 4D), lung wet/dry weight ratio (Fig. 4E), IL-1β and MIP-2 concentrations (Fig. 4F, 4G), and ameliorated lung histological changes (Fig. 4H, 4I).

## Discussion

The goal of the present study was to determine whether HMGB1 was involved in the exacerbation of moderate tidal volume MV on LPS challenged lung and explore the underlying mechanism. The main finding was that moderate tidal volume MV significantly augmented pulmonary inflammatory response and increased IL-1β, IL-6, TNF-α, MIP-2 and HMGB1 release induced by LPS *in vivo*. Synergistic upregulation of pulmonary capillary permeability and cellular responses was attenuated by HMGB1 blocking antibody. *In vitro* experiment showed that cyclic stretch induced HMGB1 expression through pathways that include p38 MAPK and NF-κB.

Previous studies have shown that moderate tidal volume MV itself did not cause extensive lung injury in normal lung, but augmented lung inflammatory responses to pre-injured lungs [28], [29]. Although most studies showed no increase of cytokines such as TNF-α and MIP-2 with moderate tidal volume MV, a more pronounced increase in these cytokines was observed when combined with other injurious strategies such as LPS [6], [30]. In accordance with these studies, we showed here that moderate tidal volume ventilation did not independently induce the production of IL-1β, IL-6, TNF-α and MIP-2 but did increase their concentrations in BALF when combined with LPS. A clinical study indicated that MV was associated with increased HMGB1 concentrations in BALF [17]. Importantly, lung injury caused by ventilation was mitigated by HMGB1 antibody [18]. It is known that not only HMGB1 released passively by ruptured or dead cells but also secreted by cells that have been activated by a variety of stimulus, including LPS, TNF-α and mechanical stretch [19], [31], [32]. Extracellular HMGB1 also contributes directly to the production of proinflammatory cytokines and development of ALI [33]. Thus, HMGB1 might play an important role in ALI. In the present study, we confirmed that MV, either alone or in combination with LPS, regulated HMGB1 expression in lung. Importantly, treatment with HMGB1 antibody significantly ameliorated lung injury, suggests that MV exacerbated LPS induced lung injury is mediated by HMGB1.

It is known that mechanical forces activate several intracellular signal pathways, including the MAPKs family and NF-κB [34]–[37]. Our recent study indicated that mitogen activated protein kinase kinase 6, an upstream kinase of p38, involved in mechanical stretch induced HMGB1 expression [20]. The *in vitro* finding is supported by the present *in vivo* finding that MV and LPS independently activated MAPKs. Furthermore, MV significantly enhanced LPS induced MAPKs activation. However, we noticed that treatment with SB203580 but not PD98059 and SP600125 attenuated HMGB1 expression, indicating that mechanical stretch increased HMGB1 expression might due to p38 instead of ERK and JNK. NF-κB is another key factor involved in VILI. A previous *in vitro* study showed that NF-κB activation correlated with elevated levels of IL-8 induced by mechanical stretch [38]. Ning et al. found that mechanical stretch can activate p65 translocation and IκB-α degradation, thus inducing the secretion of IL-8 [39]. In an *in vivo* experimental model, blockage of NF-κB decreases VILI [40]. We show here that MV and LPS each induced the degradation of IκB-α, MV enhanced LPS induced IκB-α degradation. NF-κB factor p65, which translocated from cytoplasm to nucleus by LPS challenge, was also strongly augmented by MV. Importantly, pretreatment with NF-κB inhibitor blocked mechanical stretch induced HMGB1 expression following LPS challenge.

**Figure 5:**
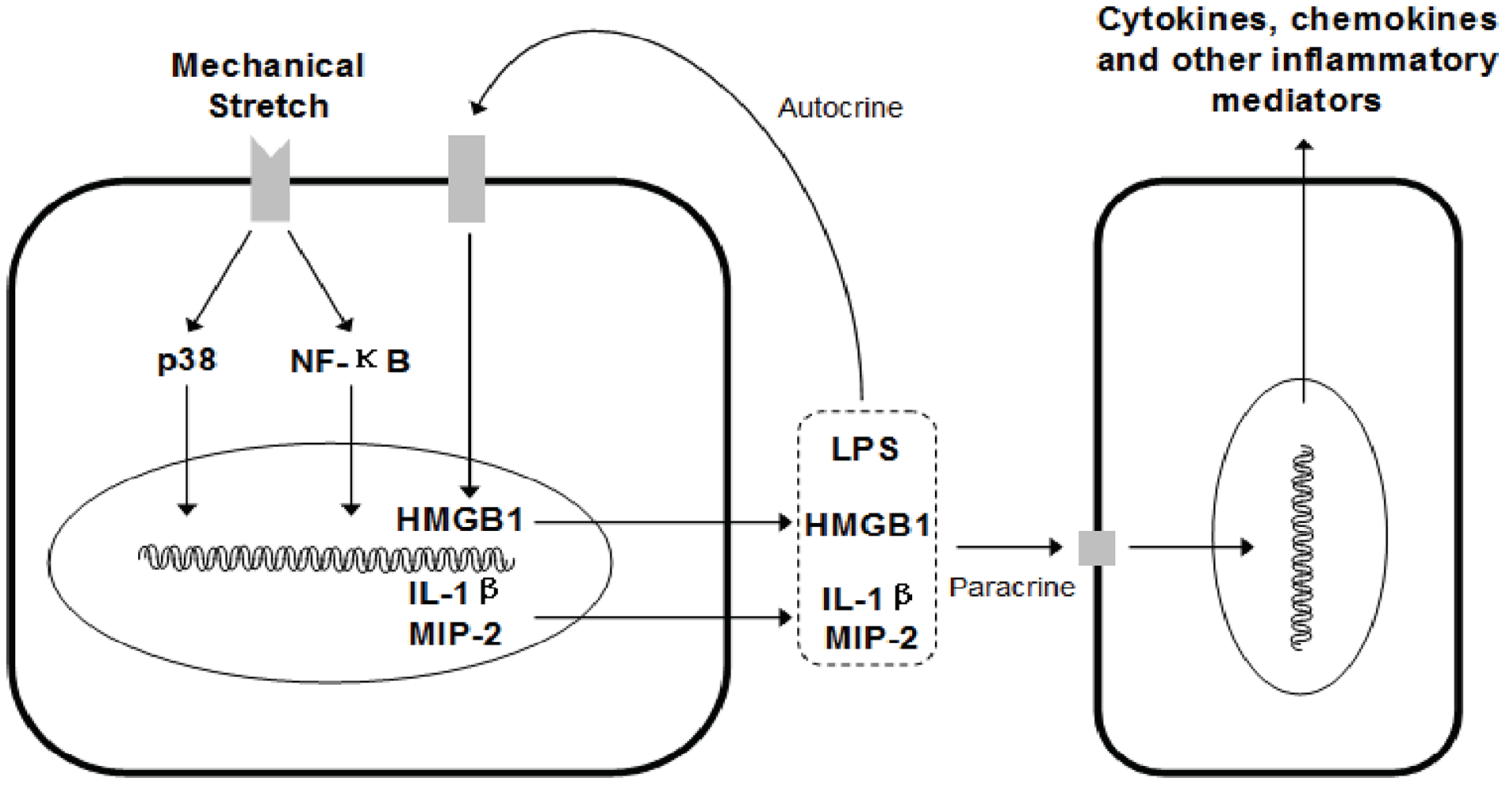
Proposed mechanism of HMGB1 in sustaining inflammatory response in ventilator-induced lung injury. Mechanical stretch is sensed by mechanosensor apparatus and activates p38 MAPK and NF-κB cascades, initiating HMGB1 genes transcription. Nuclear HMGB1 then translocates to the cytoplasm and subsequent being released into the extracellular milieu. The extracellular HMGB1 forms complexes with other molecules (e.g. LPS, IL-1β) and such complexes in turn activate the cells. This forms a positive autocrine/paracrine feed back loop, which prolongs and expands the process of VILI.

Altemeir and colleagues [41] evaluated transcriptional responses to MV, LPS, and the combination of MV and LPS using gene microarrays with a murine model of lung injury. They found that when MV was combined with intratracheal LPS there was broad augmentation of gene transcription, which was associated with enhanced inflammation and the development of ALI. Included within the highly up-regulated genes were chemokines, cytokines, transcription factors, components of the MAPK cascade, and other genes associated with lung injury. By using systemic injection of LPS instead of intratracheal instillation, we established a model that mimics the systemic inflammatory response of sepsis. As expected, the augmented cytokines production and lung inflammation were also observed in our investigations, and we further demonstrated the involvement of HMGB1. Although extracellular HMGB1 acts as a proinflammatory cytokine [42], recent studies demonstrated that purified HMGB1 had no proinflammatory activity. Instead, it bound a number of pathogen-associated molecules and enhanced the cytokine effects of these molecules [21]–[25]. Here, we propose that HMGB1 itself induced by mechanical stretch dose not produce pro-inflammatory activity, but may interact with LPS or other cytokines and potentiate their pro-inflammatory effect. This also well explains our observation that MV alone induced HMGB1 expression without significant changes of cytokines and lung histology; however, a synergistic interaction between MV and LPS was presented for cytokines production and lung injury. Interestingly, the concentration of HMGB1 in BALF was strikingly correlated with IL-1β, IL-6 and MIP-2, strongly supported our hypothesis that HMGB1 plays a pivot role in the development of VILI, and suggested that upregulation of HMGB1 during VILI might be either contributor of other cytokines production or consequence of cytokines insult. Thus, HMGB1, accompanied by LPS and/or IL-1β, induced the production of cytokines including HMGB1 and IL-1β themselves, suggesting a role for HMGB1 in promoting inflammation through an autocrine/paracrine feedback loop (Fig. 5).

There were also some limitations in the present study. For example, the animals were ventilated with room air with 10 ml/kg tidal volume and zero PEEP. This ventilation setting might not be an adequate ventilatory management for patients with acute lung injury. In patients with sepsis-induced lung injury, a protective MV mode, including a low tidal volume (4–8 ml/kg) and FiO_2_-PEEP combinations (usually FiO_2_≥0.5 and PEEP≥8 cmH2O), generally be employed. However, for this study, our data support the idea that moderate tidal volume ventilation enhanced LPS induced HMGB1 expression.

## Conclusions

Moderate tidal volume MV alone did not cause extensive lung injury in normal lung, but augmented pre-existing lung injury. However, the mechanism remains unknown. Our data demonstrate that moderate tidal volume ventilation enhanced LPS induced HMGB1 expression, which is associated with the severity of inflammatory lung injury. The mechanisms are, at least in part, through activation of p38 MAPK and NF-κB pathway. More investigations on the proinflammatory activities of HMGB1, especially its interaction with other cytokines, will likely lead to new understanding for mechanisms and developing potential protective interventions of VILI.
